# Reminiscenses of Hawaii

**Published:** 1913-08

**Authors:** John C. Warbrick

**Affiliations:** Chicago


For Texas Medical Journal.

### Reminiscenses of Hawaii.—En Route for the Pahaha Sugar Plantation.

BY JOHN C. WARBRICK, M. D., CHICAGO.

Leaving the pretty Kealakekua Bay, not a very important place of call, and Captain Cook's monument behind, we steamed on further down the coast. Soon after the boat got well under way Baker began to prepare a new kind of a mixture, which he said was very good for a drink. It consisted of the milk of one or two of the cocoanuts he brought on board mixed with some of the gin he had with him, the latter having some kind of a diuretic action. This mixture, he seemed to think, was good for a number of things, such as to relieve headache, settle one's stomach or to cure seasickness. Not very long afterwards he had a good chance to find out whether it had any value or not in case of the latter. A little was more than enough, and it did not turn out to be such a good remedy as he expected, and more so for seasickness, for which it seems hardly anything you can mention does very much good.

As we were passing a small place on the coast called Kauhako the boat suddenly received a heavy jolt by striking something head-on in the water, from which it seemed to rebound just for a moment. This caused some alarm among the passengers and made them think the boat had received serious injuries and was going to sink. However, their fears were soon quieted by an officer of the vessel, who said there was no cause for alarm. It was almost certain the boat struck a huge shark in its course, as they are to be found in large numbers in that part. A little further along the coast several of them could be readily seen in a group close to the shore as if basking quietly in the hot sun with their ugly horns projecting just above the surface of the water. The thought of having to face one of those terrible monsters of the deep was revolting, for it could be safely said at any time no one was anxious to have such an experience. It is stated that some of the Kanakas or natives have been known to defeat a shark they are such expert swimmers and performers in the water, but 10 to 1 on the shark would be a safe bet at any time.

Sailing quietly on we came to Okae, and between it and Ka Lae it was of some interest to see where the numerous lava flows from the side of the great white mountain Mauna Loa took place from time to time on that side of the crater, while a number of flows occurred on the opposite side also. Mauna Loa is about 16,000 feet high, while some of the lava flows from it have extended for miles over the country, and as they did carry along everything in their track, uproot large trees, easily destroy houses and cause ruination on all sides. One flow, it is said, ran a long distance into the sea, extending the shore line for more than a mile and killing thousands of fish, which floated on the surface of the water afterwards.

As we got near a place called Honnapo the weather was windy and the water rough, causing a number of people to be seasick, and among them Baker. He felt bad and wished more than once he had never taken any of his cocoanut mixture, for instead of settling his stomach it only gave it a nice upset, so he was anxious to get off the boat as quick as possible. More than once he could be heard giving the long regulation heave over the side of the vessel, so well known to those who are seasick and who suffer intensely for the time being. Baker would gladly have accepted any help he could get, but there seemed to be nothing that could be done to relieve him, except to get to land, almost the surest remedy in most cases.

Punalun, the next stopping place, was reached towards evening, and as the boat anchored some distance out the water continued rough, while it drizzled rain, so landing in the small boats was made only with difficulty. We decided to spend the night at this place, for Baker being rather used up needed sleep and rest, so we went to the only hotel in the place to refresh ourselves with a night's rest before starting our journey up the country the next morning. Leaving orders for a good breakfast and to be called early, we soon turned in. Sleep that night was almost impossible, even under well-protected mosquito netting, for with the numerous cat quarrels that had to be settled, yelping dogs and troublesome mosquitoes, about the only thing to do was to lie awake. Up early the next morning, we had breakfast and were soon ready to leave after paying our bills. Baker's account read bed and breakfast $1.00, while mine was the same fare, $2.00. Baker got off easier because he was going to remain in that district for a time, while the proprietor knew he would never see me again on account of going thousands of miles away, so he could charge what he liked and nothing would be said about it. Business is often dull in some of those out-of-the-way places. Proprietors are often glad to get a chance to lay for the right persons whenever they happen along. The weather was very hot as we left Punalun by train to go up the country. The railway was just a narrow gauge line of two feet six inches, used mostly for plantation purposes, as hauling merchandise from Punalun on the coast to the Pahala Sugar Plantation, about six miles inland. We took seats on a small open flat car to view the surrounding country, as the grade was uphill and the train went slow. In about an hour we were at the large Pahala sugar mills, and this was the destination of Baker, where he had to get his first experiences of plantation life. A delay of two hours had to be made here before the guide who carried the mail could be ready to leave for the volcano house. In the meantime Baker had a chance to look over the quarters he had to live in for the next few months, if he could survive that long. This was just a small single room on the ground floor at one end of a low board building longer than broad, one window with small panes of glass on the outside, and an old dusty couch in one corner, a small mirror on the wall and a creaky old chair. This was about all the comfort there was to be had, and it seemed kind of raw to Baker, and not very inviting. It was, however, rough and ready; early and late; hard and strong in plantation life. Parlor tactics were not allowed in that part. Tender hands and soft words did not get the work done, so the prospects did not look as if things were going to be smooth and easy for Baker, and he wondered how he was going to get along, for he had never been much accustomed to the strenuous life at any time, and more so on a sugar plantation, nor did he ever live so far apart from his friends before.

